# Seasonal variation in medico-legal autopsies in Finland—a nationwide analysis of the period 2016–2021

**DOI:** 10.1007/s00414-022-02880-4

**Published:** 2022-08-30

**Authors:** Petteri Oura

**Affiliations:** 1 Department of Forensic Medicine, Faculty of Medicine, University of Helsinki, P.O. Box 21 (Haartmaninkatu 3), Helsinki, FI-00014 Finland; 2 Forensic Medicine Unit, Finnish Institute for Health and Welfare, P.O. Box 30 (Mannerheimintie 166), FI-00271 Helsinki, Finland

**Keywords:** Seasonality, Trend, Variation, Medico-legal autopsy, Finland

## Abstract

Both natural and unnatural mortality have seasonal variation. In spite of the established link between season and mortality, it is unclear whether medico-legal autopsies are subject to similar variation. Building on a nationwide dataset from the years 2016–2021, this short report aimed to analyse whether medico-legal autopsies are subject to seasonal variation in Finland. An electronic information system was queried for the monthly numbers of performed autopsies. Monthly and yearly trends were estimated with Kruskal–Wallis test and linear regression. A total of 50,457 medico-legal autopsies were performed during the 6-year study period. There were on average 29 to 47 autopsies per day, with an estimated annual decline of 1.8% (95% confidence interval 0.7–2.9%) over the study period. Monthly and yearly variation in autopsies was mostly minor and irregular; statistically significant differences were only observed between January and September as well as January and November (*p* < 0.05). As such, there appears to be little seasonal variation in medico-legal autopsies in Finland. A mild declining trend in the number of autopsies was observed. Future studies are invited to explore patterns of seasonality in other medico-legal systems, for example in those with generally lower autopsy rates than in Finland.

## Introduction

Mortality is subject to seasonal variation [1–5]. Not only does this concern natural causes such as cardiovascular death (peaks during heat waves and cold spells) [6, 7], but also unnatural causes such as drowning (peak in summer) [8, 9], hypothermia (peak in winter) [10], and suicide (various peaks depending on population) [11, 12]. Environmental and behavioural factors, e.g. weather and holidays, have been suggested as potential drivers of seasonality [1, 4].

In spite of the established link between season and mortality, it is unclear whether medico-legal autopsies are subject to similar variation. Currently, there is a paucity of reports addressing seasonality in the context of medico-legal autopsies. This information would be important not only for administrative purposes, i.e. resource allocation and scheduling, but also for detecting warning signals of emerging health and safety concerns.

In Finland, a police-led investigation is mandatory in all deaths that involve suspicion of homicide, suicide, accidental death, medical or surgical adverse event, or occupational disease, and when the death is sudden or unexpected [13]. The investigation generally includes a medico-legal autopsy. All medico-legal autopsies are performed under the same authority, namely, the Finnish Institute for Health and Welfare, in five regional offices. Approximately 15% of total deaths undergo a medico-legal autopsy. Building on a comprehensive nationwide dataset from the years 2016–2021, this short report aimed to analyse whether medico-legal autopsies are subject to seasonal variation in Finland.

## Materials and methods

This was a retrospective register-based analysis, utilising monthly numbers of medico-legal autopsies in Finland between 2016 and 2021. The data were released by the Finnish Institute for Health and Welfare which constitutes the only authority in charge of medico-legal autopsies nationally. Ethical approvals were not required because the study was based on summary data.

In 2016, a nationwide electronic information system (OLT) was established for comprehensive medico-legal documentation. The system maintains records of all medico-legal autopsies performed nationally. For this short report, the OLT system was queried for the monthly numbers of performed autopsies over the period 2016–2021.

As medico-legal autopsies are generally performed on business days, a special statistic was needed to allow comparisons across months with varying numbers of business days. To serve this purpose, the total number of autopsies in a month was divided by the number of business days in the corresponding month (“autopsies per day”).

Statistical analysis was performed in SPSS version 27 (IBM, Armonk, NY). Monthly and yearly medians as well as the minima and maxima of performed autopsies (autopsies per day) were illustrated by box plots. Differences between years and months were analysed by Kruskal–Wallis test in a pairwise manner with post hoc Bonferroni correction. Additionally, monthly data were plotted consecutively over the entire study period (January 2016 to December 2021), and a trend was analysed using linear regression with 95% confidence interval (CI) for the slope. *p* values < 0.05 were considered statistically significant.

## Results

**Fig. 1:**
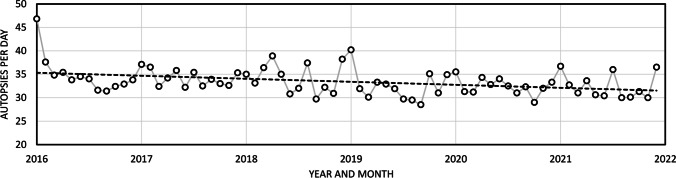
Monthly trends in medico-legal autopsies across the study period. Dashed line indicates a linear trend; an annual decline of 1.8% (95% CI 0.7–2.9%) occurred over the period

A total of 50,457 medico-legal autopsies were performed in Finland during the 6-year study period (Table 1). Monthly visualisation of autopsies per day is presented in Fig. 1. There were on average 29 to 47 autopsies per day, with an estimated annual decline of 1.8% (95% CI 0.7–2.9%) over the study period. Fluctuations between months were mostly minor and irregular.

**Table 1:** Total numbers of medico-legal autopsies performed in Finland over the years 2016–2021

Year	Number of autopsies
2016	8799
2017	8580
2018	8551
2019	8137
2020	8201
2021	8189
All	50,457

**Fig. 2:**
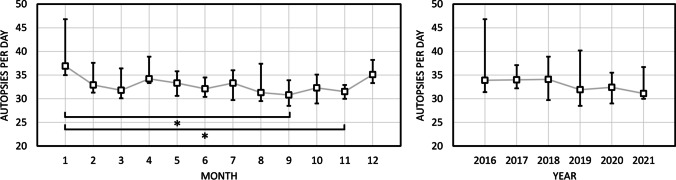
Monthly (left) and yearly (right) variation in the number of medico-legal autopsies per business day. Boxes indicate median values and whiskers indicate minimum and maximum values over the study period. Square brackets indicate statistically significant difference between categories (*p < 0.05)

Monthly and yearly variation in autopsies per day are illustrated in Fig. 2. As for monthly variation, the year-end period (December to January) showed moderately higher rates than the remaining months; however, statistically significant differences were only observed between January and September as well as January and November. There were no significant differences between the rest of the months, or between any of the years.

## Discussion

This short report of 50,457 medico-legal autopsies in 2016–2021 aimed to analyse seasonal variation in medico-legal autopsies in Finland. Two main conclusions stemmed from the data. Firstly, a mild but statistically significant decline occurred in the number of autopsies performed per day over the study period. Secondly, there was little monthly and yearly variation in medico-legal autopsies.

Clinical autopsies have followed a declining trend for a relatively long time, both internationally [14] and domestically [13]. Even though the Finnish medico-legal autopsy rate remains high, it has been undergoing a mild decline over the recent decade. This is an expected response to a national policy change where the actual need of medico-legal autopsy has been more strictly monitored [13]. According to the present analysis, this mild decline has continued also in 2016–2021.

Although all-cause mortality and unnatural mortality have seasonal variation [1], the present analysis found little monthly and yearly variation in medico-legal autopsies. Only the differences between January and September as well as January and November were statistically significant. Year-end periods appeared to be slightly busier in terms of autopsies than the remaining months. In general, however, the present data do not support the presence of regular major monthly or yearly variation in Finnish medico-legal autopsies.

Speculatively, the lack of association could be explained by lack of seasonal patterns in underlying mortality; however, there are reports confirming that at least some causes of death show clear seasonal variation also in Finland [8, 15]. Another explanation may be the high medico-legal autopsy rate in Finland, which may overwhelm the potential seasonal effects. Opposing seasonality patterns (e.g. lower number of suicides but higher number of unintentional drownings in the summer) may also obscure each other, with a net effect close to zero. To the best of the author’s knowledge, there is lack of previous reports addressing seasonality in medico-legal autopsies. Future studies are encouraged to model cause-specific seasonality, making use of individual-level datasets. It would also be important to explore whether patterns of seasonality exist in other medico-legal systems, for example in those with generally lower autopsy rates.

The strengths of this short report include nationwide approach and high coverage of medico-legal autopsies performed over the study period. The main limitations include retrospective design and summary-level data with no background information of the deaths.

In conclusion, there was little monthly and yearly variation in medico-legal autopsies in Finland over the period 2016–2021. A mild declining trend in the number of autopsies continued. Future studies are invited to explore patterns of seasonality in other medico-legal systems, for example in those with generally lower autopsy rates than in Finland.

## Data Availability

The dataset used in this study is available from the corresponding author on reasonable request.

## References

[1] Bierton C, Cashman K, Langlois NEI (2013). Is sudden death random or is it in the weather?. Forensic Sci Med Pathol.

[2] Wu Y, Li S, Zhao Q (2022). Global, regional, and national burden of mortality associated with short-term temperature variability from 2000–19: a three-stage modelling study. Lancet Planet Health.

[3] Madaniyazi L, Armstrong B, Chung Y (2022). Seasonal variation in mortality and the role of temperature: a multi-country multi-city study. Int J Epidemiol.

[4] Falagas M, Karageorgopoulos D, Moraitis L (2009). Seasonality of mortality: the September phenomenon in Mediterranean countries. CMAJ.

[5] Ledberg A (2020). A large decrease in the magnitude of seasonal fluctuations in mortality among elderly explains part of the increase in longevity in Sweden during 20th century. BMC Public Health.

[6] Abrignani MG, Lombardo A, Braschi A (2022). Climatic influences on cardiovascular diseases. World J Cardiol.

[7] Töro K, Bartholy J, Pongrácz R (2010). Evaluation of meteorological factors on sudden cardiovascular death. J Forensic Leg Med.

[8] Lunetta P, Smith GS, Penttilä A, Sajantila A (2004). Unintentional drowning in Finland 1970–2000: a population-based study. Int J Epidemiol.

[9] Girela-López E, Beltran-Aroca CM, Dye A, Gill JR (2022). Epidemiology and autopsy findings of 500 drowning deaths. Forensic Sci Int.

[10] Brändström H, Eriksson A, Giesbrecht G et al (2012) Fatal hypothermia: an analysis from a sub-arctic region. Int J Circumpolar Health 71:18502. 10.3402/IJCH.V71I0.18502 10.3402/ijch.v71i0.18502 PMC3417546 22584518

[11] Lasota D, Pawłowski W, Krajewski P (2019). Seasonality of suicides among victims who are under the influence of alcohol. Int J Environ Res Public Health.

[12] Kaya A, Tosun Tasar P, Meral O (2020). The characteristics of older people suicides by sex and age subgroups. Leg Med (Tokyo).

[13] Kuvaja P, Pakanen L, Alajärvi S (2018). How the cause of death is established in Finland. Suom Lääkäril.

[14] Burton JL, Underwood J (2007). Clinical, educational, and epidemiological value of autopsy. Lancet.

[15] Ryti NRI, Mäkikyrö EMS, Antikainen H (2017). Risk of sudden cardiac death in relation to season-specific cold spells: a case–crossover study in Finland. BMJ Open.

